# Introducing medical students with social aspects of old age

**DOI:** 10.1093/geroni/igaf122.3613

**Published:** 2025-12-31

**Authors:** Yehudit Reuveni

**Affiliations:** The university of Haifa, Haifa, Hefa, Israel

## Abstract

During the second year of medical school at the “Ruth and Bruce Rappaport Faculty of Medicine” in the Technion, students participate in workshops that also introduce social aspects of old age. Workshops are conducted and held by a teamwork and leadership expert and usually take place in an assisted-living facility, also can be framed as “protecting housing”, in which independent individuals choose to live during their elderhood. Within the workshop, students get to meet the residents, have conversations with them, and then, in small groups, act together as committees that has to propose recommendations to improve health services for the elderly. During this activity, students are exposed to strengths and practical wisdom gained over the years, and are witnessing the bright side of elderhood, through which they get a real opportunity to reframe aging, to reframe and withdraw agist attitudes, and build a more age-friendly professional identity.

